# Malaria infection and severe disease risks in Africa

**DOI:** 10.1126/science.abj0089

**Published:** 2021-08-20

**Authors:** Robert S Paton, Alice Kamau, Samuel Akech, Ambrose Agweyu, Morris Ogero, Charles Mwandawiro, Neema Mturi, Shebe Mohammed, Arthur Mpimbaza, Simon Kariuki, Nancy A Otieno, Bryan O Nyawanda, Amina F Mohamed, George Mtove, Hugh Reyburn, Sunetra Gupta, Philip Bejon, José Lourenço, Robert W Snow

**Affiliations:** 1 Department of Zoology, University of Oxford, Oxford, UK; 2 Kenya Medical Research Institute (KEMRI) - Wellcome Trust Research Programme, Nairobi, Kenya; 3 Centre for Tropical Medicine and Global Health, Nuffield Department of Clinical Medicine, University of Oxford, Oxford, UK; 4 Eastern and Southern Africa Centre of International Parasite Control, Kenya Medical Research Institute (KEMRI), Nairobi, Kenya; 5 Kenya Medical Research Institute (KEMRI) - Wellcome Trust Research Programme, Kilifi, Kenya; 6 Child Health and Development Centre, Makerere University, College of Health Sciences, Kampala, Uganda; 7 Kenya Medical Research Institute (KEMRI) - Centre for Global Health Research, Kisumu, Kenya; 8 Kilimanjaro Christian Medical Centre/Joint Malaria Programme, Moshi, Tanzania; 9 London School of Hygiene and Tropical Medicine, London, UK; 10 National Institute for Medical Research, Amani Research Centre, Muheza, Tanzania

**Keywords:** Malaria, *Plasmodium falciparum*, severe malaria, anaemia, cerebral malaria, Africa

## Abstract

The relationship between community prevalence of *Plasmodium falciparum* and the burden of severe, life-threatening disease remains poorly defined. To examine the three most common severe malaria phenotypes from catchment populations across East Africa, we assembled a dataset of 6506 malaria admissions in children aged 3 months to 9 years from 2006 to 2020. Admissions were paired with data from community parasite infection surveys. A Bayesian procedure was used to calibrate uncertainties in exposure (parasite prevalence) and outcomes (severe malaria phenotypes). Each 25% increase in prevalence conferred a doubling of severe malaria admission rates. Severe malaria remains a burden predominantly among young children (3 to 59 months) across a wide range of community prevalence typical of East Africa. This study offers a quantitative framework for linking malaria parasite prevalence and severe disease outcomes in children.

*Plasmodium falciparum* causes one of the most deadly, preventable parasitic diseases in Africa. Our understanding of the relationship between parasite exposure, clinical immunity, and malaria mortality is limited by lack of empirical evidence. The attribution of malaria deaths in Africa continues to rely on sparse, outdated information derived from interviews with bereaved relatives (1–3). These methods lack sensitivity and specificity in the absence of confirmatory clinical and parasitological examinations (4). Modelled interpolations on predicted age patterns of malaria mortality have been met with skepticism (5). An alternative source of information is age patterns of severe, potentially life-threatening malaria admissions to hospitals; we use these data to investigate the relationship between community prevalence of *Plasmodium falciparum* and the burden of severe, life-threatening disease.

During the 1990s and early 2000s, ecological analyses were undertaken at various hospital sites to define the age-specific rates of hospitalization from communities with known levels of parasite exposure (6) or proxies of transmission intensity based on temperature-related effects of altitude on transmission (7). Age and clinical phenotypes of severe malaria varied by transmission intensity, such that disease incidence declined rapidly in young children in areas of high transmission in which disease presentations were dominated by severe malaria anaemia (SMA). By contrast, disease incidence declined more slowly with age under conditions of low transmission intensity. Under such conditions, cerebral malaria (CM) was proportionally more common than in areas of high transmission. A historical controversy (8–11) regarding the effects of altering natural parasite exposure, immunity, and the medium- to long-term impacts of vector control and chemoprevention (12–15) was reignited by the observation that across a wide range of transmission settings common to Africa at that time, overall rates of severe malaria among children aged <10 years appeared similar.

Despite major increases in the coverage of control activities across Africa (16) and a changing landscape of infection prevalence (17), there have been no large-scale epidemiological descriptions of the rates of severe malaria among African children in the past 20 years. Understanding how changes in community parasite prevalence alters the rate and age distribution of children hospitalized with severe malaria is essential for optimizing and predicting the impact of malaria control efforts. Given the scarcity of detailed time series data that indicate how interventions lower transmission and reduce the malaria burden (18), comparing and contrasting data patterns (e.g., age patterns) from sites with different malaria ecologies presents an opportunity to infer what transitioning between different transmission regimes might represent in terms of the rate and age distribution of severe malaria cases.

To define the incidence of pediatric severe malaria admissions against community-based levels of parasite prevalence (age-standardised *Plasmodium falciparum* parasite rate, *Pf*PR_2-10_), we analysed active surveillance data from 13 hospitals in East Africa. These hospitals served 26 communities over 35 time-site specific periods spanning 2006 to 2020 (supplementary materials section 1, fig. S1, and table S1) in which community-based malaria parasite prevalence was recorded (supplementary materials section 1 and table S2). The dataset contains 924 months of hospital observations and 833,864 child-years of community risk across the 35 time-specific catchment areas between 2006 and 2020. A total of 6506 malaria admissions in children aged 3 months to 9 years were used to define severe malaria. The 35 time-site locations represent the range of contemporary malaria ecologies common to the subregion, from historically negligible transmission as determined by parasite prevalence (*Pf*PR_2-10_ <1%) at Kabale (Uganda) to high transmission sites (*Pf*PR_2-10_ >67%) at Bungoma, Busia, Siaya (Kenya), and Muheza (Tanzania) (table S2).

The rates of three common severe malaria phenotypes–severe malaria anaemia (SMA), respiratory distress (RD) and cerebral malaria (CM–were modelled for each of the 35 time-site periods. A Bayesian regression model was implemented with propagated uncertainty in parasite prevalence (supplementary materials section 2.1) and syndrome reporting (supplementary materials section 2.2) to define the relationship between the time-matched, age-standardized diagnostic-corrected parasite prevalence and minimum community-adjusted severe malaria rates per 1000 children per annum (p.a.) (supplementary materials section 1). For a given time-site, the number of malaria admissions for each severe phenotype (or for all three combined) was modelled with three model forms: intercept-only, log-linear, and three-parameter log-logistic models. These model forms were compared using the difference in model deviance information criterion (ΔDIC) (19) to test the hypothesis that severe malaria rates were independent of a linear or asymptotic (sigmoidal) function of community parasite prevalence. Each model form was fitted with both a Poisson and negative binomial distribution; the latter accounts for overdispersion in counts of admitted children (supplementary materials section 1). Model outputs are reported as the median of the Bayesian posterior estimates, with uncertainty described using highest density intervals (HDIs).

Admission rates of severe malaria increased log-linearly with community parasite prevalence (Fig. 1). Because admission counts were overdispersed, a negative binomial distribution was a better fit than a Poisson (ΔDIC = 1716). A log-linear form for ƒ(*Pf*PR_2-10_) was favoured over a log-logistic despite an identical DIC, because more complex functional forms with more parameters must be justified by a considerable reduction in the DIC. An intercept-only formulation performed poorly compared with the log-linear structure (ΔDIC = 22) (table S4). The selected model suggests that with every 25% increase in community parasite prevalence, annual severe malaria admission rates approximately doubled (2.06 HDI: 1.58 to 2.73). This manifested as an estimated severe malaria admission rate of 1.02 per 1000 children p.a. (HDI: 0.84 to 1.28) at 25%, 2.10 per 1000 children p.a. (HDI: 1.61 to 2.95) at 50%, and 4.33 per 1000 children p.a. (HDI: 2.67 to 7.79) at 75% community parasite prevalence (Fig. 1). The model estimated that in the absence of any prevalence (*Pf*PR_2-10_ = 0), there would be a background rate of 0.49 (HDI: 0.34 to 0.74) annual admissions of severe malaria phenotypes per 1000 children p.a. Here, *Pf*PR_2-10_ = 0 was interpreted as a scenario characterized by either low survey power in detecting parasitaemia or instances in which infection was acquired outside the study area.

We examined individual relationships between incidences of SMA, RD, CM, and community parasite prevalence. Admission rates of SMA were positively associated with community parasite prevalence (Fig. 2A). The best-fitting model described the number of SMA admissions with a negative binomial distribution (ΔDIC = 225.1), indicating overdispersion. A sigmoidal curve was the best-fitting functional form for ƒ(*Pf*PR_2-10_), with a lower DIC than either a log-linear (ΔDIC = 13.4) or an intercept-only function (ΔDIC = 39.7) (table S4). The effect of community parasite prevalence on SMA admissions was multiphasic; admission rates were very low when community prevalence was low and then increased sharply towards an asymptote. With higher parasite prevalence, the model fit had higher uncertainty, entertaining a range of asymptotic admission rates when community prevalence was high. There was a positive log-linear correlation between rates of RD (a marker of severe malaria indicating acidotic breathing) and community parasite prevalence, but with high uncertainty (Fig. 2B and table S4). Admission counts were overdispersed, with the negative binomial distribution favored for explaining the counts (ΔDIC = 525.3). There was no correlation between parasite prevalence and rare presentations of CM using any model forms (Fig. 2C), although rates were still overdispersed (ΔDIC = 104.1). CM was estimated to occur at a low, constant rate (0.19 per 1000 children p.a.) (HDI: 0.13 to 0.30) for all values of *Pf*PR_2-10_.

To explore the relationship between admissions rate, age, and parasite prevalence in more detail, a model that described admissions as continuous functions of both parasite prevalence and age was developed (supplementary materials section 2.3). This model estimated that if community parasite prevalence was lower than 15.9% (HDI: 14.1 to 17.6), a uniform distribution was an adequate description of the data (Fig. 3) - i.e., site with low transmission show little or no age dependence in severe malaria admissions (fig. S2). Determining this cutoff does not preclude age dependence in admissions below this level but may reflect a lack of statistical power to identify age dependencies as severe malaria becomes rare at low-prevalence sites. Above a community parasite prevalence of 15.9%, the peak age of admission was predicted to decrease from 15.24 months (HDI: 12.18 to 18.24) at a *Pf*PR_2-10_ of 25% to 3.32 months (HDI: 3.05 to 4.02) at 75%. This shift in peak age was concomitant with an approximate four-fold increase (4.22; HDI: 2.62-7.03) in admissions across the same change in parasite prevalence (25 to 75%).

Within our data series, Tororo, Homa Bay, and Kilifi North (fig. S1) were sampled more than once, with subsequent visits coinciding with declines in community prevalence. Data obtained from these repeatedly measured sites all support the model prediction that rapid or systematic longer-term reductions in parasite prevalence have not led to an overall increase in severe malaria incidence in children aged 5 to 9 years, relative to those aged less than 5 years. For instance, we estimated that between the first data collection period in Tororo A (2012 to 2013) and the follow-up data gathered later (2017 to 2019), community prevalence declined from 64.1% (HDI: 60.7 to 67.6) to 7.0% (HDI: 4.6 to 9.6). Severe malaria admissions were reduced from 4.97 (HDI: 4.27 to 5.49) per 1000 children p.a. to 1.02 (HDI: 0.71 to 1.27). As explored in our age-dependent model, a community parasite prevalence this low was not associated with a detectable increase in the age of admission.

We applied a statistical modeling approach to an empirical data series from 35 time-site periods to provide a data-driven analysis of the functional relationships between age, immunity, disease, and exposure. The varying age distributions of severe malaria admissions from low to high intensities of community prevalence have been described previously (14, 20–22) and are consistent with expected patterns of age, exposure, and acquisition of immunity (23, 24). In our analyses, we found a strong positive association between community parasite prevalence and the admission rate of severe malaria. Each 25% increase in community parasite prevalence conferred an approximately 2-fold increase in severe malaria admission rate, accompanied by a shift in the peak rate of admissions towards younger children. In the setting with the lowest transmission intensity, the mean age of severe malaria (43.68 months) was twice as high as that in the highest transmission settings (20.88 months), though the overall rates among children aged 3 months to 9 years were considerably lower in the low-transmission settings.

Given the declining rates of SMA and RD admissions with declining community parasite prevalence (Fig. 2, A and B) versus the apparent constant rate of CM admission rates across all transmission settings, CM inevitably accounts for an increasing proportion of severe malaria cases under low transmission. However, the rates of CM were very rare as there were only 135 cases of CM in our data, reducing the power and precision to examine the relationship between the rates of CM and parasite prevalence.

Our results show that severe, life-threatening malaria remains concentrated predominantly among younger children (3 to 59 months) regardless of transmission intensity, with a slight shift towards older children in low-transmission settings. However, this does not appear to translate to any equivalence or increased lifetime risks. The ecological analysis presented here suggests that few early lifetime infections might still confer some level of functional immunity to severe malaria before age 6 under all endemic settings (Fig. 3) (25–27). Children under 5 years continue to be the focus of disease prevention control in East Africa; SMA remains the primary severe disease phenotype requiring hospital management, and CM is a rare occurence. Under endemic range of malaria that characterizes East Africa today, the composite admission rate of all severe malaria phenotypes changed log linearly with community prevalence. Although there was no evidence of an inverse relationship or plateau in rates when parasite prevalence was high, as previously inferred (6, 14), this relationship cannot be ruled out but is unlikely given the current range of parasite prevalence. Rates of severe malaria anaemia were asymptotic at high levels of malaria transmission (*Pf*PR_2-10_ >75%). The selection of the log-logistic form over the log-linear function (ΔDIC = 13.4) makes it unlikely that this saturation in admissions is solely an artifact of underestimated rates at a few high-prevalence sites. The consistency in rates of CM across transmission settings indicates that associated clinical immunity is acquired in a different way than in other pediatric phenotypes.

Ecological analyses, such as the one presented here, come with unavoidable caveats. The data from each of the sites presented are a product of a diverse range of past transmission intensities, mitigation strategies, and population structures. This can only be resolved through detailed longitudinal data, which are rare in Africa (28, 29). We have defined parasite exposure as the experience of children in their respective communities at the time their disease profiles were defined. The epidemiology of severe malaria should be reconciled across contemporary malaria ecologies in the subregion; however, this does not capture lifetime parasite exposure. Older children may have been exposed to historical transmission that would have influenced their acquisition of a functional immune response not represented by current levels of transmission intensity. Furthermore, a mixture of endemicity and intervention could explain heterogeneity. With a few exceptions such as donor-driven subnational use of indoor residual spraying (IRS), long-lasting insecticidal nets (LLINs) remain the mainstay of the subregional effort to reduce malaria parasite prevalence in East Africa, as well as throughout the sites included in the present analysis where LLIN coverage was well below universal for children at risk (table S1). Combined attacks on the *P*. *falciparum* through increased use of LLINs, chemoprevention, and additional vector control efforts with IRS have been shown to rapidly reduce parasite prevalence (30–32). In addition, prompt diagnosis and effective treatment of early-stage disease will affect the progression and rates of severe malaria hospitalization (33). Secondary data from national household surveys of fever treatment, undertaken every 3 to 5 years, lacked the precision necessary to accurately reflect the influence of early treatment on malaria hospitalization at the spatial and temporal scale of our study. To address this limitation, high spatiotemporal survey data will be required in future models relating transmission intensity to rates of severe malaria in Africa. Additionally, the number of severe disease cases reported here may not be representative of all cases, as an unknown proportion of life-threatening infections may not reach the formal health care system. This study was conducted in populations with easy geographic access to hospitals and as such the results may not be generalizable to other settings in which there is limited access to hospital care. Even so, we have provided a quantitative framework for linking community transmission intensity of malaria and its manifestation in the form of severe disease outcomes in children.

## Supplementary Material

Supplementary material

## Figures and Tables

**Figure 1:**
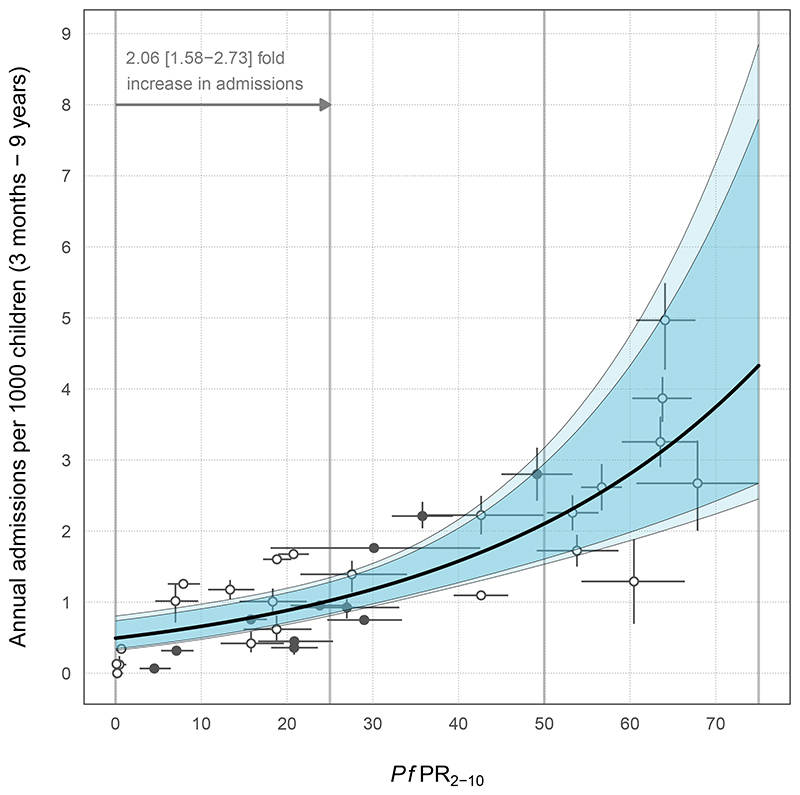
Relationship between the rate of admissions of severe malaria (combination of severe malaria anaemia, respiratory distress and cerebral malaria) and community parasite prevalence (*Pf*PR_2-10_). The median fit for the Bayesian regression model is denoted by the thick black line, with 95% and 99% highest density intervals in dark and light blue, respectively. Gray points and vertical 95% HDIs denote the model-estimated admission rates; intervals were not plotted for site periods in which a formal diagnoses of malaria phenotypes were available for all patients. The conditions of admitted malaria patients without a specific diagnosis of SMA, RD, or CM were stochastically diagnosed in submodels on the basis of other indicative symptoms. Horizontal intervals represent the uncertainty in parasite prevalence calculated from a model that age-standardizes parasite prevalence to the 2-to-10-year age range while accounting for sample size and correcting for the diagnostic method (corrected rapid diagnostic test (RDT) surveys are indicated by closed points, microscopy by open circles).

**Figure 2:**
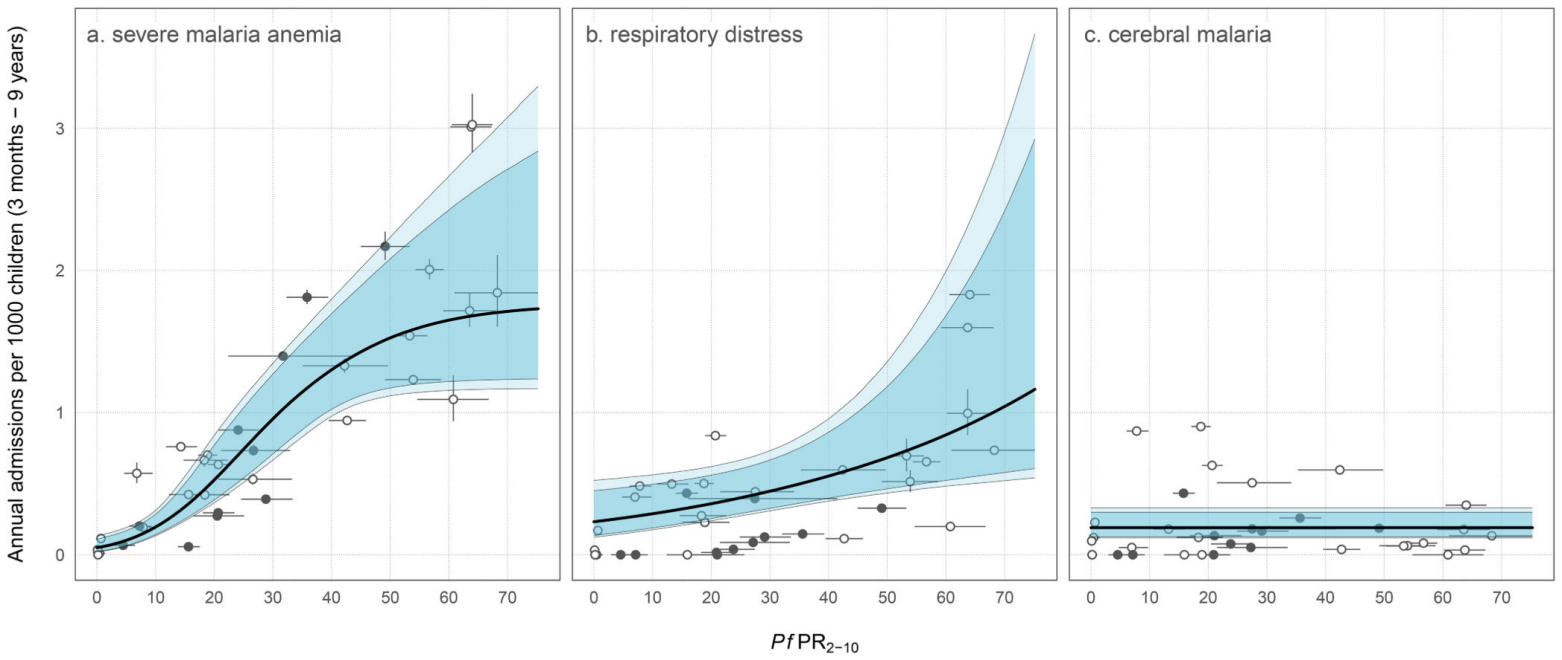
Relationship between the admission rate of individual severe malaria phenotypes and community-based parasite prevalence. Median regression model fits are denoted by the thick black line, with 95% and 99% highest density intervals in dark and light blue, respectively. **(A)** Nonlinear relationship between rates of SMA and *Pf*PR_2-10_. **(B)** shows the log-linear relationship between rates of RD and *Pf*PR_2-10_. **(C)** constant rate of CM for all values of *Pf*PR_2-10_. We modelled uncertainty in the admission rates of severe malaria anaemia and respiratory distress using a method akin to that used for the composite measure in Fig. 1. Because there were no alternative definitions of CM, uncertainty in admission rates was not considered (and therefore no vertical intervals were present). Uncertainty in community parasite prevalence was standardized to the 2-to-10-year age range, with a further correction for diagnostic method (corrected RDT surveys are shown as closed points, microscopy as open points).

**Figure 3:**
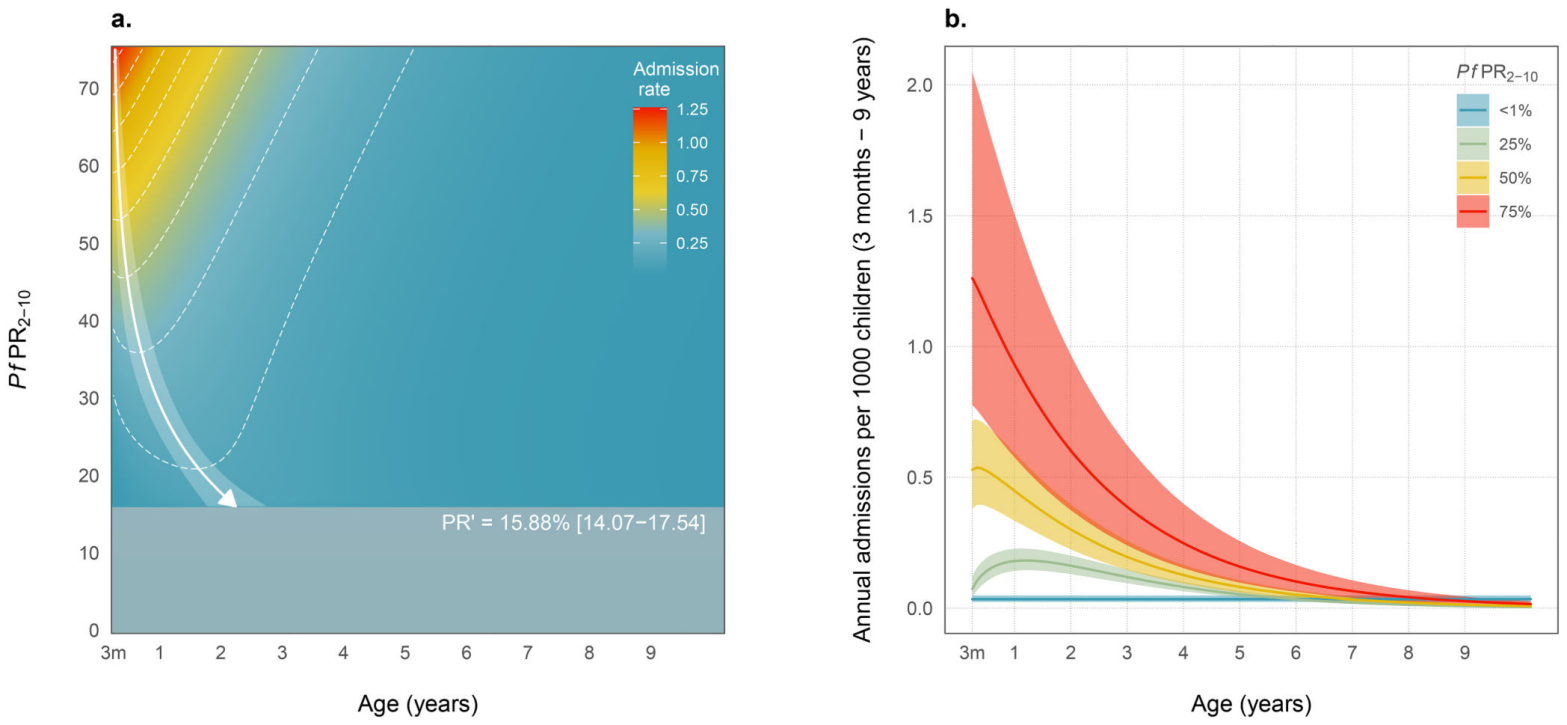
Changes in age-specific admission rate per 1000 children p.a. of severe malaria phenotypes with parasite prevalence. **(A)** Prediction surface for the model estimated rates of severe malaria for each month of childhood between 3 months and 9 years The white arrow and 95% highest density intervals show the estimated increase in the most frequent age of admission with decreasing parasite prevalence. The dark blue rectangle denotes the estimated cutoff below a parasite rate of ~ 15.9% (14.1 to17.6), below which admissions are relatively rare and can be adequately described as stochastic outcomes with age. It should be noted that this cutoff is sensitive to the amount of data available to characterise the age distribution of rare events; it is possible that our dataset lacks the requisite sensitivity to detect age dependence in severe malaria admissions at lower parasite rates. The most frequent age of admission increases with parasite prevalence, whereas the concomitant reductions in malaria incidence largely offset the shift of the burden onto other age classes. For each 25% increase in community *Pf*PR_2-10_, the age-dependent model predicted a doubling of admissions (2.05; HDI: 1.62 to 2.65); this agrees with the estimated increase for the age invariant model (2.06; HDI: 1.58 to 2.73). **(B)** The surface shown in (A) is presented as binned community parasite prevalence, including the associated 95% HDIs. The age dependence in admissions of severe malaria was modelled as a continuous process using a gamma distribution; the same diagnostic and community parasite rate submodels used in the age invariant model were also used here. A random effect for each site (on the admission rate) helped to account for sites with anomalously high or low rates of severe malaria for the corresponding estimate of community parasite prevalence.

## Data Availability

Data used in this analysis has been curated and uploaded to the Harvard Dataverse (34). Correspondence and requests for materials should be addressed to the KEMRI Wellcome Data Governance Committee (dgc@kemri-wellcome.org). These data are available through a formal requesting process to the KEMRI Institutional Data Access/Ethics Committee. Guideline details can be found on the KEMRI Wellcome website (https://kemri-wellcome.org/about-us/#ChildVerticalTab15). All codes associated with the current submission can be found on GitHub (https://github.com/abj0089/Malaria_Project/tree/1.0) and Zenodo (35).

## Summary

The risk and age-pattern of severe malaria can be predicted by the quantity of parasite exposure in a given community.
