# Supplementary material for: Malaria infection and severe disease risks in Africa
Source: Science. Author manuscript; Available in PMC 2021 Aug 31. (PMC7611598; doi:10.1126/science.abj0089)
Supplement: Supplementary material [file EMS133135-supplement-Supplementary_material.pdf]

Science
AAAS

Supplementary Materials for

Malaria infection and severe disease risks in Africa

Robert S Paton*, Alice Kamau*, Samuel Akech, Ambrose Agweyu, Morris Ogero, Charles Mwandawiro, Neema Mturi, Shebe Mohammed, Arthur Mpimbaza, Simon Kariuki, Nancy A Otieno, Bryan O Nyawanda, Amina F Mohamed, George Mtove, Hugh Reyburn, Sunetra Gupta, Philip Bejon, José Lourenço, Robert W Snow

*Correspondence to: robert.paton@zoo.ox.ac.uk and akamau@kemri-wellcome.org

This PDF file includes:

Materials and Methods
Supplementary Text
Figs. S1 to S4
Tables S1 to S5

1 MATERIALS AND METHODS

Site selection: Hospital sites were selected as those with continuous paediatric ward surveillance established for the purposes of monitoring acute febrile illness aetiology, disease burden epidemiology or research settings for improvements in hospital case-management (fig. S1; table S1). Each site used malaria diagnostics on all febrile presentations, an electronic medical record form that recorded standardised features of severe malaria (*36)* and documented residential addresses on all admissions.

Hospital catchment populations: Residential data for each admission was matched to the smallest possible area, defined using national census, located within 30 km of the hospital but excluding urban areas (fig. S1; table S1). Population counts among the selected catchments were derived from the most contemporary national census and projected forwards or backwards using district level intercensal growth rates. Age-structures of each population were corrected to single year age groups 3-11 months to 9 years by applying rural household age structures provided for the nearest time-regional matched demographic household survey data. Age-specific person years of observation (PYO) were adjusted for the months of observation included in each temporal series. The exceptions were three sites in Kilifi (Kenya) where actual household continuous population surveillance data (*37)* were used to define age specific PYO. Catchments were selected to avoid competition with other admission facilities. Our estimated hospital admissions, however, represent a minimum measure of the true community burden of severe disease, as severe events may have occurred outside of hospital, with the child either recovering or dying at home.

Community parasite prevalence: Each hospital catchment was paired with a community parasite prevalence survey. Spatially and temporally disaggregated community-based household parasite prevalence survey data were used at six time-site periods in Kenya (Kilifi and Siaya) and four time-site periods in Uganda (Tororo and Jinja 2012-2013). Surveys of malaria parasite prevalence among school children were established during or within 4 months of the paediatric ward surveillance in western Kenya 2014-2019 (Busia, Bungoma, Homa Bay, Kakamega and Vihiga) and Uganda 2017-2019 (Tororo, Apac, Mubende, Kabale and Jinja). Published estimates of parasite prevalence at three sites in Muheza, Tanzania were extracted from literature (table S2). To standardize parasite prevalence across sites, we implemented a version of the conversion algorithm by Smith et al. (2007) (*38)* which standardising parasite surveys to the 2-10 years age range (supplementary section 2.1). This, and all subsequent analyses, were conducted in the statistical software R (*39)*, with models fitted using the Bayesian inference software JAGS (*40)*. Parameter estimates were presented as median values with 95% highest density intervals (HDI) as a measure of uncertainty. Markov Chain Monte Carlo (MCMC) simulations of model parameters were run across a minimum of 6 chains until each parameter had an effective sample size of at least 10,000 and convergence had been achieved (psrf statistic < 1.02).

Malaria diagnosis: Malaria admissions were defined following a review by discharging clinicians of all available clinical, laboratory and radiological information. Children aged less than 3 months were excluded as data were incomplete, the influence of maternal immunity complicates the analysis, and severe malaria incidence is very low below 3 months. Children with underlying conditions that may have precipitated admission were excluded, these included, sickle cell disease, HIV, tuberculosis, malignancies, trauma, epilepsy, poisoning, snake/animal

bites and measles. Children with other coincidental secondary diagnoses were retained accepting that co-morbidity was common, or hard to define as primary, secondary or co-primary (*41)*. Three common paediatric phenotypes of disease severity (*36, 42, 43)* were classified from those admitted with a malaria diagnosis: severe malaria anaemia, respiratory distress and cerebral malaria. Between-site comparisons can be challenging, owing to differences in the completeness of symptom reporting and differences in clinical definitions. We therefore assessed the predictive performance of alternative definitions of these severe pathologies, using patients records where alternative symptoms were reported in combination with a diagnosis of severe disease using a Bayesian model (detailed below).

Severe malaria anaemia (SMA): SMA was formally diagnosed as a haemoglobin on admission or during the in-patient stay of less than 5 g/dl. At 16 hospital time-site periods haemoglobin level was available for > 90% of all admissions. At the remaining 19 hospital time-site periods where haemoglobin level was not reported, "severe pallor" or blood transfusion were considered as suitable proxies for SMA. A Bayesian regression framework was used to determine the predictive performance of these alternative SMA case definitions (supplementary section 2.2.1; fig. S3a, S3d). The revised number of SMA cases at each site was calculated by augmenting those patients with a haemoglobin level with those without a formal diagnosis (table S3).

Respiratory distress (RD): Deep breathing (presumed secondary to acidosis) was defined as RD, although it was difficult to confidently determine acidosis in the absence of base-excess blood gases (*44)*. RD was defined on admission at all time-site periods except three site-periods in Siaya, Kenya (table S3). There were two alternative indicators of respiratory distress: nasal flaring and intercostal recession. Nasal flaring had very poor sample coverage therefore only intercostal recession predictive performance was assessed in a Bayesian regression framework (supplementary section 2.2.2; fig. S3b, S3d). As with SMA, adjusted counts of respiratory distress as estimated by the model were calculated at each time-site period.

Cerebral malaria (CM): The standard definition of CM is based on a Blantyre Coma Score (BCS) of less than 3. A formal documentation of BCS was made at 14 time-site periods. At 21 time-site periods that did not report BCS, the child's neurological status based on an Alert, response to Voice, response to Pain, Unconscious (AVPU) scale and reported unconsciousness were deemed equivalent to BCS <3 (table S3). As two equivalent measures of CM were widely reported, we did not model diagnosis uncertainty for this phenotype, instead we tested the robustness of CM classifications to determine if different patterns would emerge using a less strict definition of CM (supplementary section 2.2.3; fig. S4).

Composite rate of severe malaria phenotypes: To estimate a combined rate of severe malaria phenotypes (SMA, RD or CM), we utilised the two Bayesian sub-models for severe malaria anaemia and respiratory distress (supplementary section 2.2.1-2.2.2). As severe malaria phenotypes are not mutually exclusive, we modelled the SMA and RD status separately with the status of each patient as a Bernoulli trial. Therefore, the probabilities used in the diagnosis models were estimated simultaneously while the SMA/RD status of unknown patients was stochastically simulated according to the probability associated with their symptom combinations and all cerebral cases were classified deterministically. This methodology includes "coin-toss" uncertainty for individuals with no formal diagnosis, as well as uncertainty from the estimated probabilities (supplementary section 2.2.4; fig. S3c).

Model fitting: A Bayesian regression model was implemented to describe the functional relationship between the *Pf*$PR_{2\text{-}10}$ estimated by the first sub model (supplementary section 2.1) and the adjusted malaria rates from the second set of sub-models (supplementary section 2.2.1-

2.2.2). Both sub-models were estimated in conjunction with the regression, meaning uncertainty in both the dependent and independent variables were propagated to the estimated relationship between admission rates and community parasite prevalence. For a given time-site, the number of malaria admissions of a single (or combination of) phenotypes was denoted X. This count was modelled by either a Poisson distribution with rate λ ($X \sim Poisson(\lambda)$) or a negative binomial distribution re-parametrized using the probability and size arguments such that $X \sim NB\left(\frac{r}{r+\lambda}, r\right)$. The latter has a variance independent of its mean, given by $\lambda\left(1+\frac{\lambda}{r}\right)$. In both cases, λ was corrected by an offset term to account for the number of children across the time period (person-years, PY: $\log(\lambda) = f(PfPR_{2-10}) + \log(PY)$). We contrasted the intercept-only, log-linear and 3-parameter log-logistic functional forms of $f(PfPR_{2-10})$ using the difference in model deviance information criterion (ΔDIC), with reductions greater than or equal to 10 in favour of the more complex model required to support the inclusion of additional parameters (*19*).

To reflect uncertainty in the severe malaria-classified admissions (owing to additional cases from non-standard diagnoses), the estimated rate at each time-site period, λ_s, had to be scaled to account for underestimation of cases. The proportional difference between the estimated rate for a site (λ_s, which was estimated exclusively from only diagnosed cases at the site, X_s) was calculated in relation to the corrected counts estimated by the diagnosis models described above, $\tilde{X}_s$:

$$\Phi_s = \frac{\lambda_s}{\tilde{X}_s}$$

$$X_s \sim Poisson(\lambda_s \Phi_s) \quad \text{or} \quad X_s \sim NB\left(\frac{r}{r+\lambda_s \Phi_s}, r\right)$$

Here, if the diagnosis sub-model predicted no extra cases ($\tilde{X}_s = X_s$) from non-standard symptoms, Φ_s would be approximately one and the count would be modelled by λ_s. If the model predicted additional cases, then the model parameters used in the calculation of λ_s would need to be scaled up to accommodate the correction Φ_s, reflecting the fact that the empirically defined count was underestimated. This model did not adjust for environmental or intervention covariates because we regarded the estimate of community parasite prevalence as a product of these covariates in time and space.

Age-pattern of severe malaria phenotypes: To estimate the age-specific rates of severe malaria admissions, we modelled admissions as a continuous function of community parasite prevalence and age. Inter-site variation in admission rates were modelled as two interacting functions of community parasite prevalence; an overall change in the rate of admissions owing to community prevalence and a second component that described the change in the age-distribution of cases with community prevalence. As with the age-invariant model, rates were corrected for the number of person years at each site and age group, while also accounting for the previously outlined underestimation of severe malaria cases due to diagnosis uncertainty. The age distribution of admissions was assumed to be Gamma distributed, with the mode of this distribution changing as a continuous function of parasite prevalence. Based on the general age-patterns seen across the sites (fig. S2), the model also allowed for estimation of a critical parasite prevalence, *PR’*, below which the admissions with age were best described by a uniform distribution (admissions were adequately described as stochastic outcomes from an intercept rate; supplementary section 2.3; table S5).

2 Supplementary text

Our dataset had two important sources of uncertainty that we aimed to control for in the regression model of severe malaria hospital admissions against community parasite prevalence. Community parasite surveys are often undertaken across different age ranges and with different sample sizes. An algorithm developed by Smith et al. (2007) (*38)* is commonly used to standardise these estimates in the 2-10 year age range (*Pf*$PR_{2\text{-}10}$) using an epidemiologically motivated model. However, this method does not account for uncertainty in sample size or between-survey differences in epidemiological parameters. In supplementary section 2.1, a model that represents these sources of uncertainty in the estimated *Pf*$PR_{2\text{-}10}$ at each site is presented.

The second source of uncertainty was in the classification of severe malaria as either severe malaria anaemia, respiratory distress or cerebral malaria using hospital admissions data. It was difficult to standardize these diagnoses across sites since not all hospitals sites reported the precise definition of severe malaria for every case (table S3). This included missing haemoglobin measurement for potentially anaemic patients or failing to record deep breathing for patients with potential respiratory distress. Some hospitals reported alternative symptoms, such as pallor (for anaemia) while in some hospitals both standard diagnosis and other related symptoms of severe malaria were recorded. To determine the predictive performance of these alternative methods of classification, models that leveraged data from sites reporting multiple indicators for each severe malaria diagnosis were developed. These models are described in supplementary section 2.2.

Both sub-models were fed into a Bayesian regression framework. The framework incorporated the uncertainty from each sub-model in the coefficients of the model, making any inference robust to the sources of uncertainty described above. All models (both the sub-models and the regression models) were fit in R (*39)* using JAGS version 4.3 (*40)*.

2.1 Standardising community prevalence surveys

Smith et al. (2007) (*38)* developed an algorithm for standardising parasite surveys to the 2-10 years age range. This algorithm was implemented as a deterministic conversion of community parasite rates using the best fit parameters estimated from a training dataset of 121 studies. This method was subsequently extended by Gething et al. (2011) (*45)* to account for uncertainty arising from between-survey variation in sample size. A version of this procedure was implemented in this work. In brief, from the equations in Pull & Grab (1974) (*46)* we modelled the probability of being *Plasmodium falciparum* positive as an increasing function with age, *P(A)*. Let *i* denote the index study and *j* the age class (age was limited to a maximum of 85 years):

$$\mathrm{P}(A) = \mathrm{P}_{\mathrm{i}}'\left[1 - \mathrm{e}^{(-\mathrm{b}_{\mathrm{i}}\,\mathrm{A})}\right].$$

Here the parameter *b* described the rate at which the equilibrium parasite prevalence *P'* was approached with age. The sensitivity of detecting an active *P. falciparum* infection was modelled as declining beyond age *α* at rate *c* towards sensitivity *1-s*, according to the function *F(A)*:

$$\mathrm{F}(A) = \begin{cases} 1, & A < \alpha \\ 1 - s_i\left[1 - e^{-c_i(A-\alpha_i)}\right], & A \geq \alpha \end{cases}$$

The decrease in sensitivity arises either from individuals clearing bouts of malaria more quickly or supressing them entirely (*38)*. For a given survey, the functions *P(A)* and *F(A)* was scaled by the proportion of individuals in each age group. The age distribution, $S_i(A)$, was evaluated over the upper *(U)* and lower *(L)* age limits of a survey to give an estimated probability of a sampled individual being *P. falciparum* positive:

$$\rho_i = \frac{\sum_{L_i}^{U_i} P(A)F(A)S_i(A)}{\sum_{L_i}^{U_i} S_i(A)}$$

Evaluating this expression for a given age *A* gives the probability of an individual of that age being *P. falciparum* positive (in other words, ρ was the parasite prevalence for that survey). Evaluating this expression for the 2-10 age range gives an estimate of the age corrected *Pf*PR$_{2-10}$. For the parasite surveys used in our regression model, the positivity and sample sizes with age were not reported; we only had the number of people surveyed and the number *P. falciparum* positive. This prohibited the estimation of all the age-dependent model parameters from the full model used on the training dataset. Instead, we extracted the best fit parameters from Smith et al. (2007) (*38)*. This means that the estimated parasite prevalence at each site is contingent on the deterministic values derived from a set of training datasets in Smith et al. (2007) (*38)*, not the actual age distribution or parameters derived for our surveys. In other words, our HDI represent only differences in sample size, not uncertainty in the parameters of the conversion algorithm.

Using these parameters, we optimised a value of *P* (in the function *P(A)*) for each of our surveys such that the probability *ρ* best describes the number of positive samples (N_i^+) out of the total surveyed (N_i^T) between the lower (L_i) and upper (U_i) age limits of the survey:

$$\rho_i = \frac{\sum_{L_i}^{U_i} P(A)F(A)S_{i(A)}}{\sum_{L_i}^{U_i} S(A)}$$

$$N_i^+ \sim \mathrm{Binomial}(\rho_i, N_i^T)$$

This expression was evaluated within the 2-10 age limit using the estimated value of $P^`$. This gave a standardised value of *Pf*PR$_{2-10}$ for each survey for which the uncertainty in survey sample size was accounted for. These estimates are given table S2.

Surveys were conducted using different diagnostic methods for identifying parasites in blood samples (rapid diagnostic test (RDT), or microscopy). Mappin et al. (2015) *(47)* analysed a unique dataset comprised of parasite surveys matched on the basis of having a similar spatial-temporal scope but conducted using different diagnostic tests. They estimated the relationship between the parasite rate found by microscopy (PR^{MIC}) and RDT (PR^{RDT}) using the regression:

$$\phi^{-1}(PR^{MIC}) = \vartheta + \omega\, \phi^{-1}(PR^{RDT})$$

Where ϕ^{-1} was the probit function, ϑ the intercept and ω the gradient. Age-standardised parasite prevalence for RDT surveys were corrected using this equation, with priors on the parameters ϑ and ω taken from the estimates and confidence intervals reported by Mappin et al. (2015) *(47)*. It should be noted that the differences between microscopy and RDT are on the sensitivity and specificity of these tests, and that test performance may vary with age. Accounting for this was out of the scope of the data in Mappin et al. (2015) *(47)* and our study.

2.2 Modelling uncertainty in case classification

The second source of uncertainty was between-hospital variation in symptom reporting, which necessitated alternative diagnostic methods for severe malaria anaemia, respiratory distress and cerebral malaria. Models that assessed the predictive performance of non-standard symptoms for diagnosing malaria conditions were developed.

2.2.1 Severe malaria anaemia (SMA)

The standard WHO definition of SMA is haemoglobin level less than 5 g/dl. In sites where haemoglobin level was not reported, “severe pallor” recorded by the admitting clinician, or a record of a blood transfusion *(48)* (or both) could be used as an alternative indicator of SMA status. The aim was to determine the probability of correctly diagnosing a patient as having SMA while using blood transfusion and/or severe pallor as a proxy. In 2391 patients, data were available for all three parameters (i.e. the haemoglobin level, blood transfusion and severe pallor). Taking haemoglobin as the 'true' diagnosis, the predictive performance of the other measures in the absence of haemoglobin level were evaluated. The distribution of patient haemoglobin level with different classifications of pallor and blood transfusions. There was some disagreement between these proxy measures with haemoglobin level, with either false positives (severe pallor or blood transfusion would suggest anaemia when absent) or false negatives (no severe pallor or blood transfusion, but the patient is in fact anaemic). However, there were predictive merits to the alternate symptoms.

The accuracy of SMA classification using pallor and blood transfusion was explored by fitting a Binomial model to the data. The SMA status of a patient was judged by haemoglobin levels ($Hb^{+} < 5$ and $Hb^{-} \geq 5$). The number SMA positive patients ($N^{Hb^{+}}$, out of $N^{Hb^{\mathrm{T}}}$) for a given combination of symptoms was modelled as a Binomial distribution with probability π^{Hb}:

$$\mathrm{N}_{\mathrm{P,B}}^{\mathrm{Hb}^{+}} \sim Binomial\left(\pi_{P,B}^{Hb}, N_{P,B}^{Hb^{T}}\right)$$

The index *P* denoted the pallor status of a patient which was either *S* (severe) or *O* (other). The index *B* was the blood transfusion status either *T* (true) or *F* (false). There were 35 patients that were not included in this analysis because they had missing data on all the potential measure of SMA. For patients with missing haemoglobin level, the Binomial model was used to stochastically sample the status of these patients. The probabilities of the proxy measures were defined in further detail in fig. S3d. The revised number of SMA cases at each site (N^{SMA}) was calculated by augmenting those patients with a haemoglobin level ($N^{Hb^{+}}$) with those without a formal diagnosis. The number of patients with a given pallor and blood transfusion status were multiplied by the corresponding probability for their symptoms:

$$\mathrm{N}^{\mathrm{SMA}} = N^{Hb^{+}} + N_{O,F}^{Hb}\,\pi_{O,F}^{Hb} + \mathrm{N}_{\mathrm{O,T}}^{\mathrm{Hb}}\,\pi_{O,T}^{Hb} + \mathrm{N}_{\mathrm{S,F}}^{\mathrm{Hb}}\,\pi_{S,F}^{Hb} + \mathrm{N}_{\mathrm{S,T}}^{Hb}\,\pi_{S,T}^{Hb}$$

In this Bayesian framework, uncertainty in the probabilities were represented in the corrected count. fig. S3d shows the predictive performance of non-WHO definition of severe malaria anaemia from the model. The estimated SMA admissions at each site are shown in fig. S3a and compared to estimates based on the WHO categorisation alone.

2.2.2 Respiratory distress

Only one hospital in Siaya did not report respiratory distress (RD) as a marker of deep breathing. The aim was to determine whether alternative symptoms of intercostal recession and nasal flaring were predictive of RD. Nasal flaring had very poor coverage with only 81 patients reporting both deep breathing and nasal flaring. However, 3921 patients recorded both status of intercostal recession and RD. There were 75 cases with missing data on all three measures and were not included in this analysis. The correlation between nasal flaring and RD was unreliable because of reporting coverage, so it was not included in this analysis. The correlation was stronger with intercostal recession, but weaker than the correlations we found in the SMA analysis. A Binomial model was used to describe the number of deep breathing confirmed patients (N^{DB^+}) out of the total (N^{DB^+}) with probability π^{DB} based on whether they exhibited intercostal recession:

$$N_R^{DB^+} \sim Binomial\left(\pi_R^{DB}, N_R^{DB^T}\right)$$

The index *R* was used to denote the status of intercostal recession (either true, *T* or false, *F*). These estimated parameters are given in fig. S3d. A corrected count of respiratory distress at each site was evaluated:

$$N^{RD} = N^{DB^+} + N_F^{DB}\,\pi_F^{DB} + N_T^{DB}\pi_T^{DB}$$

These updated counts and associated intervals are presented in fig. S3b

2.2.3 Cerebral malaria

The standard definition of cerebral malaria is Blantyre Coma Score (BCS) of less than 3 (this corresponds to unconsciousness). In our dataset, hospitals that did not report a BCS instead reported if unconsciousness was observed or provided a score on the AVPU (Alert, Voice, Pain, Unresponsive) scale. As both alternative measures described the key characteristic of unconsciousness from the standard defined diagnosis, they were deemed fully equivalent to a BCS less than 3. We tested the robustness of CM classifications to determine if different patterns would emerge. In fig. S4, we show that a less strict definition of cerebral malaria (AVPU of U or P or a BCS < 4) did not change our conclusion that there was little support for an association between cerebral malaria cases and changing community transmission.

2.2.4 Composite of all severe malaria classifications

We also aimed to present a model of the combined rates of all types of severe malaria. Severe malaria was defined as a diagnosis with one or more of SMA, RD or CM. As before, the WHO classification was used as the gold standard (Hb < 5 g/dl, RD and BCS <3). The counts for CM, as before, included those patients with an AVPU score of "U" or an observation of unconsciousness.

Patients with incomplete records but had at least one diagnosis of severe malaria condition were considered positive in the composite metric. There were, however, many more complex cases that were accounted for. As we had no alternative diagnosis for CM (other than the

equivalents we described in supplementary section 2.2.3), missing values for these diagnoses were treated as false in the composite analysis. However, for SMA and RD, it was possible to implement the models from supplementary sections 2.2.1 and 2.2.2 simultaneously to determine the most likely composite status based on the alternative symptoms described previously. In the Bayesian framework, any patient without haemoglobin level (and therefore a missing entry for SMA status) and/or no record of deep breathing (and therefore a missing entry for RD status) were inferred based on the information provided by pallor, blood transfusion or intercostal recession. The model used the relationships estimated in supplementary sections 2.2.1 and 2.2.2 (obtaining estimates for $\pi_{P,B}^{Hb}$ and π_{R}^{DB}) and used these relationships to stochastically sample the SMA and RD status for patients according to a Bernoulli trial. Patients with at least one positive diagnosis was categorised as being positive for the composite metric. Through this, a distribution around the number of individuals who should be included in the composite metric but for whom complete records were not available was built. These estimated counts are shown in fig. S3c.

2.3 Age-dependent model of malaria admissions

We modelled the change in admission rate of severe malaria phenotypes (λ) with age, *a,* and across sites, *s*, using a multi-part function. First, the rate of admission at each site was described by a log-linear function of parasite prevalence, *f(PR,s)*. Second, these modeled rates were distributed across ages according to one of two probability distributions. Below a critical community parasite prevalence, *PR'*, the age distribution of admissions was described by a uniform function, while a Gamma distribution was used otherwise:

$$\lambda_{a,S} = f(PR, s) \times \begin{cases} PR > PR', \int_{a}^{a+\delta a} \Gamma(\kappa, \rho)\, \delta a \div \int_{a^{min}}^{a^{max}} \Gamma(\kappa, \rho)\left(a^{max} - a^{min}\right) \\ PR \le PR', \int_{a}^{a+\delta a} \text{Uniform}\left(a^{min}, a^{max}\right) \delta a \end{cases}$$

The integral is used to determine the probability of admissions falling in each of the discrete age brackets (3 months – 0.99 years, 1-1.99 years etc). Note that, for the Gamma distribution, in order for the area under the curve to sum up to one across our 3 months-9.9 year age range we scaled it by the total area under the curve between the maximum and minimum of the age range. The function *f(PR,s)* – as with the non-age-dependent model – describes the overall composite rate of admissions and is by a log-linear function of parasite prevalence (with coefficient β and intercept α), corrected for the number of person-years in that age group and site-period ($PY_{a,s}$). Sites with atypical deviations from the average rate of admission for a particular community parasite prevalence are modelled using a random effect (a per-site draw from a normal distribution, centred on zero, ε_s):

$$log\left(f(PR, s)\right) = log\left(PY_{a,s}\right) + \alpha + \epsilon_s + \beta\, PR$$

This predicted rate (*f(PR)*) is distributed across ages according to either the Gamma (with shape, κ, and rate, ρ) or a uniform probability distribution; the latter is used by the model when

admissions are rare, and age distributions are not easily characterised. Determining which distribution is optimal is decided by a parameter *PR'* and is estimated by the model. The interaction between the age distribution of cases and parasite prevalence was modelled by reparametrizing the gamma distribution by its mode, *M*, and standard deviation, σ:

$$\rho = \frac{M(PR) + \sqrt{(M(PR)^2 + 4\sigma^2)}}{2\sigma^2}$$

$$\kappa = 1 + M(PR) \times \rho$$

With the mode, *M*, a linear function of parasite prevalence, using a log-link:

$$\log(M(PR)) = \gamma + \tau\, PR$$

This allows the model to estimate the change in the most frequent age of admissions with parasite prevalence. In effect, the model decomposes the data into first, changes in admission rates and second, shifts in the age distribution of cases in response to community parasite prevalence.

As is the case with the age-invariant model, a correction ($\Phi_{a,s}$) is calculated for the rate $\lambda_{a,s}$, by diagnosing patients with no formal classification of a malaria phenotype, based on other indicative symptoms, giving a model estimated count $\tilde{X}_{a,s}$. The number of known severe malaria cases $X_{a,s}$, are then modelled as a Poisson distribution:

$$\Phi_{a,s} = \frac{\lambda_{a,s}}{\tilde{X}_{a,s}}$$

$$X_{a,s} \sim Poisson(\lambda_{a,s})$$

The fit of this model is described in fig. 3b in the main text, with the individual fits for each site given in fig. S2. Table S2 gives the estimated parameters.

3 Supplementary figures

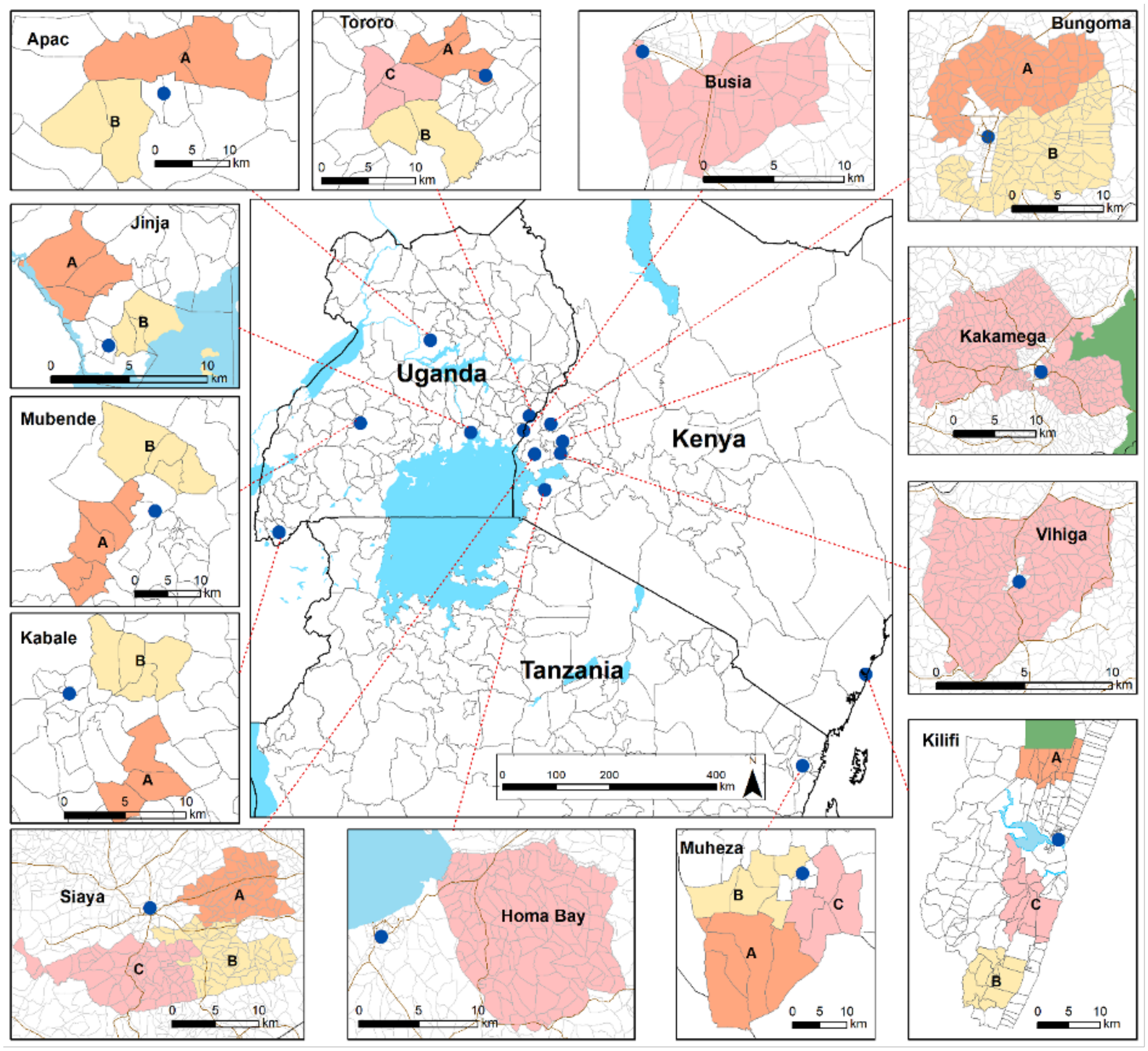

Fig. S1.
Geographical location of the 13 surveillance hospitals (blue dots) and respective census defined catchment areas (shaded pink, yellow and orange).

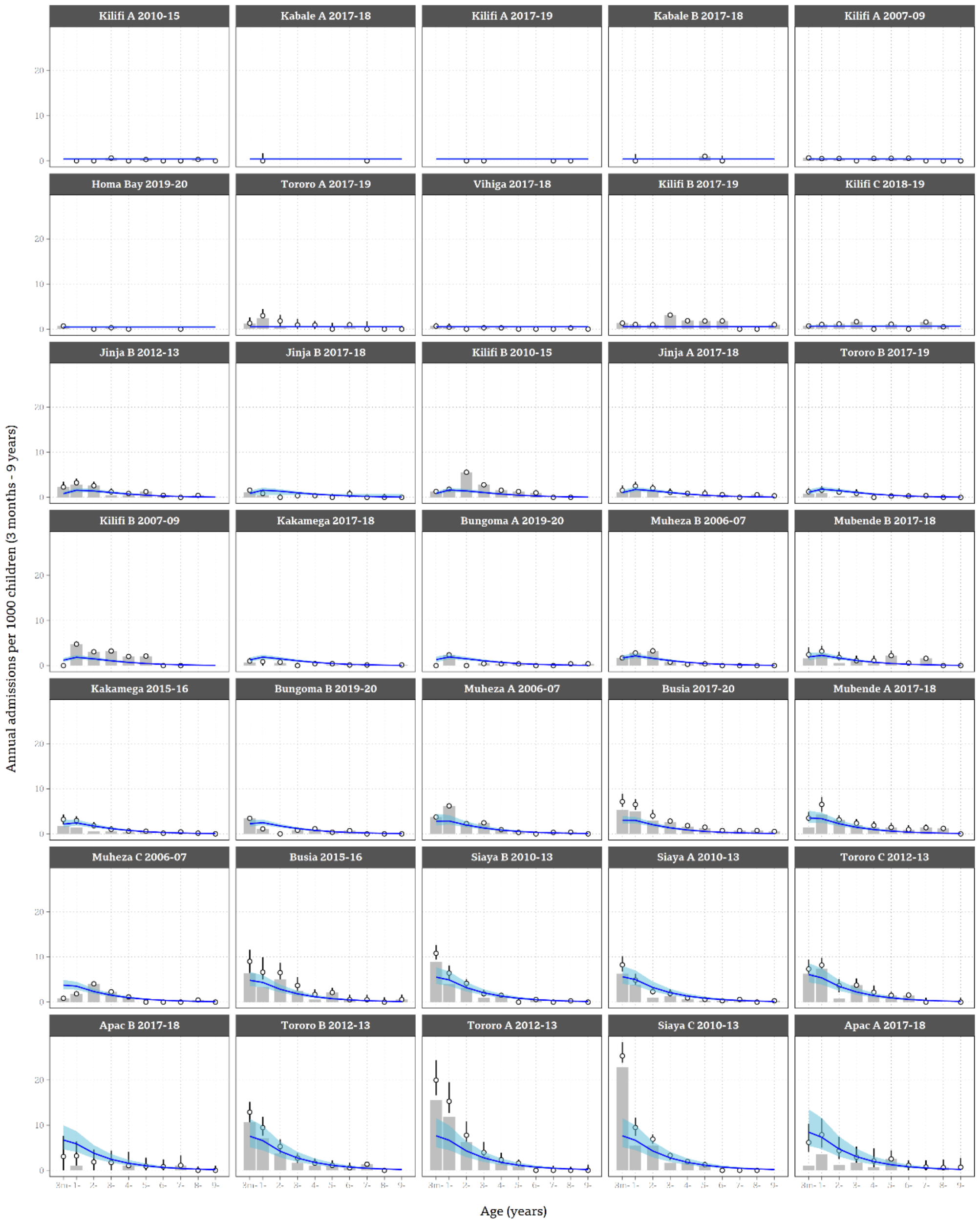

Fig. S2.

Model fits for each site period, ordered from lowest to highest estimated parasite prevalence. Blue lines and intervals give the model fit for each site, including the random effect correction on the function *f(PR),* integrated across the corresponding age bracket. White points and black intervals show the rates estimated for each age bracket in each site from the diagnosis sub-model. Grey bars give the deterministically diagnosed cases from the data, where the presence of a severe phenotypes is certain. Examples of sites with very high admissions (e.g. Siaya C 2010-2013) and atypical age distributions for the level of community transmission (e.g. Apac A 2017-2018, because of past malaria control) are not captured precisely. These sites will be reflected in the uncertainty of model coefficients (represented in Fig. 3B).

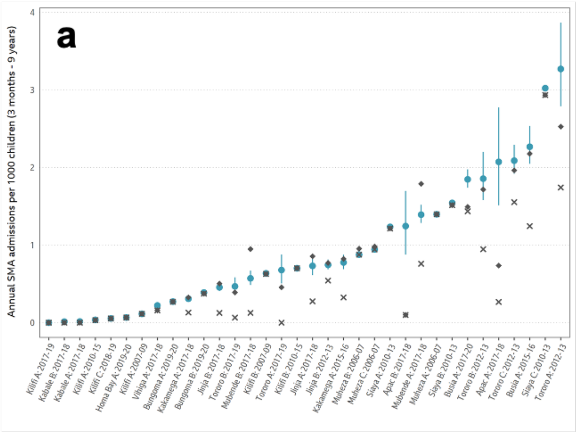

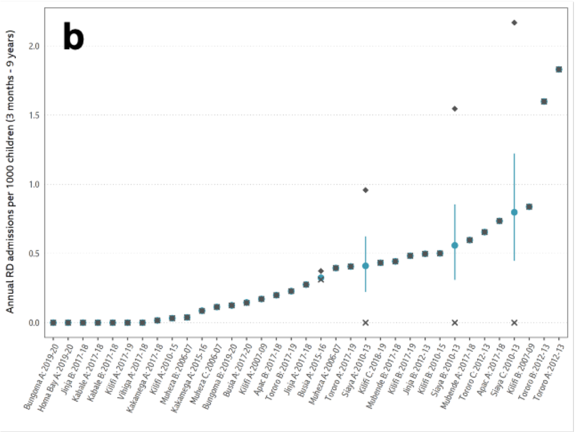

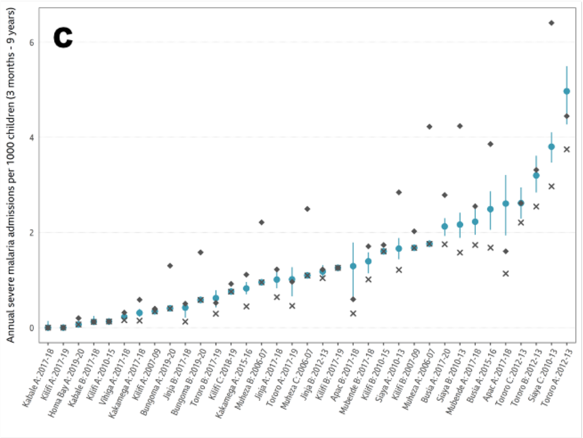

d

Parameter	Description	Lower 95% HDI	Median	Upper 95% HDI
**Severe malaria anaemia**				
$\pi_{O,F}^{Hb}$	Probability *Hb⁺* without severe pallor or a blood transfusion	0.04	0.05	0.06
$\pi_{O,T}^{Hb}$	Probability *Hb⁺* without severe pallor but received a blood transfusion	0.47	0.51	0.56
$\pi_{S,F}^{Hb}$	Probability *Hb⁺* with severe pallor but no blood transfusion	0.22	0.29	0.37
$\pi_{S,T}^{Hb}$	Probability *Hb⁺* with severe pallor and a blood transfusion	0.57	0.63	0.69
**Respiratory distress**				
π_{F}^{DB}	Probability of deep breathing without intercostal recession	0.02	0.03	0.04
π_{T}^{DB}	Probability of deep breathing with intercostal recession	0.16	0.23	0.30

Fig. S3.

Estimated rates of single and composite of severe malaria phenotypes. **(A)** are the estimated rates of severe malaria anaemia at each site are shown in blue with 95% HDIs (sites are ordered by this estimated rate). Grey crosses show the SMA rates based only on patients with the WHO-approved haemoglobin diagnosis and grey diamonds the SMA rates based on the interpretation of all three measures (pallor, blood transfusion and haemoglobin). Note that for some sites the model will not change predictions because all children had a blood sample taken. Moreover, the false negative rate when interpreting a patient with neither severe pallor nor a blood transfusion ($\pi_{O,F}^{Hb}$) will mean that the model may estimate a higher rate than the deterministic interpretation of all three measures from the data. Moreover, model estimated rates can fall lower than the deterministic interpretation of all alternative symptoms as these symptoms have an imperfect predictive relationship with SMA. **(B)** are the estimated rates of respiratory distress. The most notable adjustments are made at the Siaya hospitals where deep acidotic breathing was not reported at all; the model moderates high rates observed from if all cases of intercostal recession were taken as equivalent to deep breathing. A small adjustment is made to the rate in Busia (2015-2016) from a few cases with reported intercostal recession but no information on RD. **(C)** is the model estimated rates of the combined measure (SMA, RD and CM) of severe malaria incidence. The model moderated or increased rates at sites where accepted diagnoses are sparse. **(D)** are the parameter estimates from the model analysing the predictive performance of non-standard definitions of severe malaria phenotypes. The SMA status of a patient was judged by haemoglobin levels ($Hb^+ < 5$ and $Hb^- \geq 5$) and RD status by deep breathing.

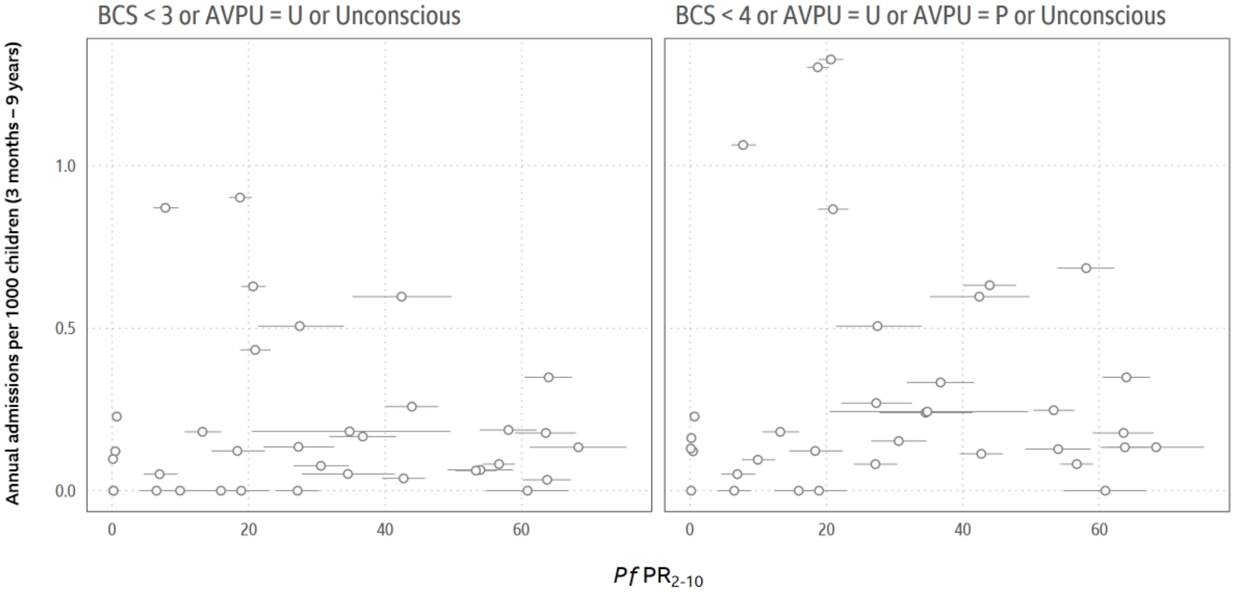

Fig. S4.
The absence of a clear association between cerebral malaria and community prevalence was robust to less stringent definitions of phenotype

4 Supplementary tables

4.1 Geographic scope

The 13 hospital sites located in East Africa serving 26 communities included in this analysis were Apac, Tororo, Jinja, Mubende, and Kabale districts in Uganda, Busia, Bungoma, Kakamega, Vihiga, Siaya, Homa Bay and Kilifi counties in Kenya and finally Muheza district in Tanzania (fig. S1). The detailed descriptions of each site over a combined 35 time-site specific periods spanning from 2006 to 2020 are shown in table S1.

Table S1.
Site specific descriptions

Site, dates (months of surveillance)	Main surveillance citation	Min-Max distance to Hospital (km)	Person-years of observation 3m-9 years [census year projected]	Insecticide net coverage in children	Indoor Residual House spraying dates
Kilifi A, Kenya<br>2007-09 (36)<br>2010-15 (72)<br>2017-19 (24)	*(44)*	9-17	17,542 [actual]<br>30,851 [actual]<br>12,361 [actual]	33% (0.5-14 years) [b]<br>48% (0.5-14 years) [b]<br>45% (0.5-14 years) [b]	None
Kilifi B, Kenya<br>2007-09 (36)<br>2010-15 (72)<br>2017-19 (24)	*(44)*	20-32	14,322 [actual]<br>29,942 [actual]<br>10,342 [actual]	74% (0.5-14 years) [b]<br>66% (0.5-14 years) [b]<br>78% (0.5-14 years) [b]	None
Kilifi C, Kenya<br>2018-19 (12)	*(18)*	6-18	18,475 [actual]	75% (0.5-14 years) [b]	None
Siaya A, Kenya<br>2010-13 (48)	*(49, 50)*	3-17	31,312 [2009]	55% (0.5-4 years) [c]	None
Siaya B, Kenya<br>2010-13 (48)	*(49, 50)*	2-18	32,331 [2009]	55% (0.5-4 years) [c]	None
Siaya C, Kenya<br>2010-13 (48)	*(49, 50)*	4-16	29,979 [2009]	55% (0.5-4 years) [c]	None
Busia, Kenya<br>2015-16 (12)<br>2017-20 (25)	*(51, 52)*	1-14	16,066 [2009]<br>34,813 [2009]	80% (4-14 years) [d]<br>67% (4-14 years) [d]	None
Kakamega, Kenya<br>2015-16 (12)<br>2017-18 (12)	*(51, 52)*	0.5-15	58,375 [2009]<br>61,300 [2009]	17% (4-14 years) [d]<br>62% (4-14 years) [d]	None
Vihiga, Kenya<br>2017-18 (12)	*(51, 52)*	0.5-17	31,527 [2009]	55% (4-14 years) [d]	None
Bungoma A, Kenya[a]<br>2019-20 (11)	*(52)*	2-16	22,268 [2009]	59% (4-14 years) [d]	None
Bungoma B, Kenya[a]<br>2019-20 (11)	*(52)*	1-14	24,027 [2009]	56% (4-14 years) [d]	None
Homa Bay, Kenya[a]<br>2019-20 (11)	*(52)*	1-21	30,088 [2009]	78% (4-14 years) [d]	2018, 2019
Jinja A, Uganda<br>2017-18 (24)	*(53, 54)*	2-9	32,713 [2014]	52% (5-14 years) [d]	None
Jinja B, Uganda<br>2012-13 (24)<br>2017-18 (24)	*(53, 54)*	1-9	22,103 [2014]<br>23,828 [2014]	36% (0.5-14 years) [c]<br>57% (0.5-4 years) [d]	None

Site, dates (months of surveillance)	Main surveillance citation	Min-Max distance to Hospital (km)	Person-years of observation 3m-9 years [census year projected]	Insecticide net coverage in children	Indoor Residual House spraying dates
Tororo A, Uganda<br>2012-13 (24)<br>2017-19 (36)	*(53, 54)*	0.2-9	11,474 [2014]<br>19,697 [2014]	56% (0.5-14 years) [b]<br>47% (5-14 years) [d]	2015, 2016, 2017, 2018, 2019
Tororo B, Uganda<br>2012-13 (24)<br>2017-19 (36)	*(53, 54)*	6-14	16,894 [2014]<br>30,693 [2014]	67% (0.5-14 years) [b]<br>40% (5-14 years) [d]	2015, 2016, 2017, 2018, 2019
Tororo C, Uganda<br>2012-13 (24)	*(53, 54)*	5-14	10,225 [2014]	34% (0.5-14 years) [b]	2015, 2016, 2017, 2018, 2019
Apac A, Uganda<br>2017-18 (21)	*(53, 54)*	2-15	14,964 [2014]	23% (5-14 years) [d]	2010, 2011, 2012, 2013, 2014; stopped; one round 2017
Apac B, Uganda<br>2017-18 (21)	*(53, 54)*	3-17	10,066 [2014]	24% (5-14 years) [d]	2010, 2011, 2012, 2013, 2014; stopped; one round 2017
Mubende A, Uganda<br>2017-18 (24)	*(53, 54)*	3-19	18,430 [2014]	47% (5-14 years) [d]	None
Mubende B, Uganda<br>2017-18 (24)	*(53, 54)*	4-18	15,802 [2014]	36% (5-14 years) [d]	None
Kabale A, Uganda<br>2017-18 (22)	*(53, 54)*	6-13	7,206 [2014]	86% ((5-14 years) [d]	2006, 2007
Kabale B, Uganda<br>2017-18 (22)	*(53, 54)*	2-11	8,526 [2014]	90% (5-14 years) [d]	2006, 2007
Muheza A, Tanzania<br>2006-07 (12)	*(55)*	9-33	32,913 [2012]	28% (0.5-4 years) [c]	None
Muheza B, Tanzania<br>2006-07 (12)	*(55)*	0.5-22	26,215 [2012]	28% (0.5-4 years) [c]	None
Muheza C, Tanzania<br>2006-07 (12)	*(55)*	2-17	26,427 [2012]	28% (0.5-4 years) [c]	None

a. Surveillance data not used from April 2020 due to alterations in service use as a result of the COVID19 pandemic
b. Long-lasting insecticide treated net (LLIN) use determined from community-based household surveys within the catchment areas during the surveillance period
c. LLIN use defined during school surveys undertaken within the catchment area during the surveillance period
d. LLIN use defined for the wider district that the catchment area was located in during national demographic and health surveys, or malaria indicator household surveys undertaken close to the surveillance period.

4.2 Assembly of parasite prevalence data

Parasite prevalence surveys undertaken as either household surveys within each catchment community (CS) or school surveys (SS) spanning from 2006 to 2019 are described in detail in table S2.

Table S2.
Site specific community-based parasitological surveys

Site, dates	Dates Community (CS) or School Survey (SS)**	Positive/examined [Age range, years]	*Pf*PR$_{2-10}$ % [95% HDI]¶	Citation
Kilifi A, Kenya				
2007-09	2007-09 (CS)	10/1645 [0.5-14.9]	0.68 [0.32, 1.12]	
2010-15	2010-15 (CS)	1/1439 [0.5-14.9]	0.12 [0, 0.34]	*(56, 57)*
2017-19	2017-19 (CS)	0/355 [0.5-14.9]	0.2 [0, 0.87]	
Kilifi B, Kenya				
2007-09	2007-09 (CS)	406/2061 [0.5-14.9]	20.75 [18.96, 22.55]	*(56, 57)*
2010-15	2010-15 (CS)	404/2261 [0.5-14.9]	18.82 [17.2, 20.5]	
2018-19	2017-19 (CS)	62/841 [0.5-14.9]	7.91 [6.09, 9.84]	
Kilifi C, Kenya				
2018-19	2018-19 (CS)	267/1336 [0.5-14.9]	**15.78 [13.9, 17.66]**	*(18)*
Siaya A, Kenya				
2010-13	2010-13 (CS)	234/454 [0.1-14.9]	53.81 [49.12, 58.65]	*(58)*
Siaya B, Kenya				
2010-13	2010-13 (CS)	588/1155 [0.1-14.9]	53.29 [50.3, 56.33]	*(58)*
Siaya C, Kenya				
2010-13	2010-13 (CS)	492/808 [0.1-14.9]	63.79 [60.26, 67.2]	*(58)*
Busia, Kenya				
2015-16	2014 (SS)	330/596 [4-14.9]	**49.16 [45.01, 53.28]**	Unpublished
2017-20	2019 (SS)	285/681 [4-14.9]	**35.79 [32.22, 39.34]**	
Kakamega, Kenya				
2015-16	2014 (SS)	65/198 [4-14.9]	**26.97 [21.25, 33.09]**	Unpublished
2017-18	2018-19 (SS)	204/789 [4-14.9]	**20.82 [18.14, 23.59]**	
Vihiga, Kenya				
2017-18	2018-19 (SS)	56/596 [4-14.9]	**7.1 [5.3, 9.09]**	Unpublished
Bungoma A, Kenya				
2019-20	2019 (SS)	77/297 [4-14.9]	**20.85 [16.63, 25.36]**	Unpublished
Bungoma B, Kenya				
2019-20	2019 (SS)	137/392 [4-14.9]	**29 [24.69, 33.4]**	Unpublished
Homa Bay, Kenya				
2019-20	2019 (SS)	24/397 [4-14.9]	**4.52 [2.81, 6.44]**	Unpublished
Jinja A, Uganda				
2017-18	2019 (SS)	68/400 [5-16.9]	18.33 [14.46, 22.29]	*(52)*
Jinja B, Uganda				
2012-13	2012-13 (CS)	80/637 [0.5-14.9]	13.36 [10.8, 16.18]	*(52, 59)*
2017-18	2019 (SS)	59/400 [5-16.9]	15.8 [12.26, 19.64]	
Tororo A, Uganda				
2012-13	2011-13 (CS)	501/820 [0.1-14.9]	64.08 [60.7, 67.63]	*(52, 59)*
2017-19	2019 (SS)	27/425 [5-16.9]	6.98 [4.64, 9.64]	
Tororo B, Uganda				
2012-13	2011-13 (CS)	303/499 [0.1-14.9]	63.54 [59.05, 67.94]	*(52, 59)*
2017-19	2019 (SS)	70/399 [5-16.9]	18.78 [14.93, 22.86]	
Tororo C, Uganda				
2012-13	2011-13 (CS)	994/1836 [0.1-14.9]	56.7 [54.29, 59.05]	*(60)*
Apac A, Uganda				
2017-18	2019 (SS)	127/199 [5-16.9]	67.87 [60.75, 74.95]	*(54)*

Site, dates	Dates Community (CS) or School Survey (SS)**	Positive/examined [Age range, years]	*Pf*PR$_{2-10}$ % [95% HDI]¶	Citation
Apac B, Uganda 2017-18	2019 (SS)	167/294 [5-16.9]	60.46 [54.34, 66.4]	*(54)*
Mubende A, Uganda 2017-18	2019 (SS)	77/195 [5-16.9]	42.63 [35.56, 49.9]	*(54)*
Mubende B, Uganda 2017-18	2019 (SS)	53/208 [5-16.9]	27.57 [21.56, 33.99]	*(54)*
Kabale A, Uganda 2017-18	2019 (SS)	0/400 [5-16.9]	0.18 [0, 0.79]	*(54)*
Kabale B, Uganda 2017-18	2019 (SS)	1/400 [5-16.9]	0.44 [0.01, 1.25]	*(54)*
Muheza A, Tanzania 2006-07	2008 (CS)	13/39 [0.1-4.9]	**30.14 [18.08, 42.53]**	*(61)*
Muheza B, Tanzania 2006-07	2008 (CS)	164/671 [0.1-99.0]	**23.82 [20.41, 27.37]**	*(62)*
Muheza C, Tanzania 2006-07	2008 (CS)	417/1050 [0.4-19.9]	42.63 [39.44, 45.79]	*(62)*

** At each site sampling strategies varied. In Siaya A-C and Kilifi A-B households were sampled annually as part of long-term surveillance; Kilifi C, 4 rounds of household sampling were undertaken to correspond with the hospital surveillance period. School surveys were undertaken in Busia, Kakamega, Vihiga Bungoma, Homa Bay in Kenya and Jinja A, Jinja B 2017-2018, Tororo A 2017-2019, Tororo B 2017-2019, Apac A & B, Mubende A & B, and Kabale A & B in Uganda. The universe of all public, primary schools located in the hospital catchment areas were included in the survey rounds and annual community-based household surveys were undertaken. Community surveys undertaken in Jinja B and Tororo A and C in 2012-2013 were part of wider household sample surveys undertaken across the respective districts, and only data from the catchment parishes were included. Village level data were identified from published findings at the three sites in Muheza, Tanzania to reflect time-site transmission estimates in these sites. The different sampling approaches precludes the inclusion of sampling weights in the estimate of *Pf*PR$_{2-10}$, however at all sites, excluding Muheza, the samples covered the entire community or school catchment areas within the selected hospital catchment areas.

¶ Parasite prevalence is corrected to the 2-10 age range as described in supplementary section 2.1. Estimates given in bold were conducted by RDT and were corrected to a microscopy value using a regression framework given in Mappin et al. (2015) *(47)*.

4.3 Assembly of hospital data

Malaria clinical data was obtained from 13 hospitals. The number of malaria admissions and the indicators for each severe malaria diagnosis are summarized in table S3.

Table S3.
Hospital surveillance clinical indicator definition

Site, dates	Malaria admissions	BCS/APVU (missing)	Severe malaria anaemia based on Hb or C (missing)	RD based on DB or ICNF (missing)
Kilifi A, Kenya				
2007-09	22	BCS (1)	HB (0)	DB (0)
2010-15	23	BCS (0)	HB (2)	DB (0)
2017-19	4	BCS (0)	HB (0)	DB (0)
Kilifi B, Kenya				
2007-09	96	BCS (0)	HB (2)	DB (0)
2010-15	139	BCS (0)	HB (0)	DB (0)
2017-19	27	BCS (0)	HB (0)	DB (0)
Kilifi C, Kenya				
2018-19	50	BCS (0)	HB (1)	DB (0)
Siaya A, Kenya				
2010-13	306	APVU (0)	HB (3)	ICNF (1)
Siaya B, Kenya				
2010-13	379	APVU (1)	HB (8)	ICNF (2)
Siaya C, Kenya				
2010-13	510	APVU (0)	HB (11)	ICNF (2)
Busia, Kenya				
2015-16	264	APVU (13)	C (0)	DB (9)
2017-20	388	APVU (43)	C (2)	DB (33)
Kakamega, Kenya				
2015-16	355	APVU (9)	C (0)	DB (12)
2017-18	132	APVU (2)	C (0)	DB (1)
Vihiga, Kenya				
2017-18	71	APVU (8)	C (7)	DB (8)
Bungoma A, Kenya				
2019-20	84	BCS (0)	HB (2)	DB (1)
Bungoma B, Kenya				
2019-20	147	BCS (0)	HB (3)	DB (0)
Homa Bay, Kenya				
2019-20	10	BCS (0)	HB (0)	DB (0)
Jinja A, Uganda				
2017-18	280	U (2)	C (1)	DB (0)
Jinja B, Uganda				
2012-13	94	U (0)	C (0)	DB (0)
2017-18	70	U (0)	C (0)	DB (0)
Tororo A, Uganda				
2012-13	350	U (2)	C (3)	DB (1)
2017-19	193	U (10)	C (8)	DB (3)
Tororo B, Uganda				
2012-13	301	U (0)	C (0)	DB (0)
2017-19	174	U (14)	C (12)	DB (8)
Tororo C, Uganda				
2012-13	142	U (0)	C (0)	DB (0)
Apac A, Uganda				
2017-18	486	U (4)	C (1)	DB (1)
Apac B, Uganda				
2017-18	209	U (3)	C (1)	DB (0)
Mubende A, Uganda				
2017-18	146	U (0)	C (0)	DB (0)
Mubende B, Uganda				
2017-18	97	U (0)	C (0)	DB (0)
Kabale A, Uganda				
2017-18	3	U (0)	C (0)	DB (0)
Kabale B, Uganda				
2017-18	3	U (0)	C (0)	DB (0)
Muheza A, Tanzania				
2006-07	473	BCS (2)	HB (0)	DB (0)
Muheza B, Tanzania				
2006-07	231	BCS (1)	HB (0)	DB (0)
Muheza C, Tanzania				
2006-07	247	BCS (2)	HB (0)	DB (0)

Cerebral malaria (Blantyre Coma Score (BCS), formal assessment of Alert, Pain, Voice and Unconscious state on admission (APVU) or observed as unconscious on admission (U); severe malaria anaemia based on haemoglobin < 5 g/dl (HB) or a composite (C) based on Haemoglobin, blood transfusion status and presence of severe pallor on admission; respiratory distress (RD) based on deep acidotic breathing (DB) or combination of intercostal recession and nasal flaring (ICNF) on admission. Figures in brackets represent numbers of admissions where clinical feature not documented for classification.

Table S4.

Parameter estimates from the Bayesian regression models described in the main text

Phenotype	functional form	$f(Pf\ PR_{2-10})$	Parameter estimates
Composite	Log-linear	$log(\lambda) = \alpha + \beta \times Pf\ PR_{2-10} + log(PY)$	α = -7.61 [-7.98, -7.22]<br>β = 2.90 [1.84, 4.01]<br>r = 0.80 [0.47, 1.20]
Severe malaria anaemia	Log-logistic	$log(\lambda) = \alpha + \frac{\beta}{1 + e^{-\gamma \times Pf\ PR_{2-10}}} + log(PY)$	α = -13.51 [-14.93, -12.08]<br>β = 7.20 [5.70, 8.71]<br>γ = 7.94 [4.68, 11.85]<br>r = 0.95 [0.55, 1.60]
Respiratory distress	Log-linear	$log(\lambda) = \alpha + \beta \times Pf\ PR_{2-10} + log(PY)$	α = -8.37 [-8.91, -7.70]<br>β = 2.14 [0.65, 3.83]<br>r = 0.31 [0.18, 0.49]
Cerebral malaria	Intercept only	$log(\lambda) = \alpha + log(PY)$	α = -8.57 [-8.97, -8.12]<br>r = 0.81 [0.45, 1.46]

Each model has an estimated value of the parameter r as the negative binomial model was the best fitting in all cases.

Table S5.
Parameter estimates from the age-dependent model of admission rates.

Parameter	Lower 95%	Median	Upper 95%
β	1.93	2.88	3.90
α	-5.87	-5.50	-5.05
γ	1.32	1.86	2.48
τ	-10.28	-7.34	-5.14
σ	2.13	2.25	2.38
PR' (%)	14.07	15.88	17.54
